# Older Adults—Potential Users of Technologies

**DOI:** 10.3390/medicina55060253

**Published:** 2019-06-07

**Authors:** Vita Lesauskaitė, Gytė Damulevičienė, Jurgita Knašienė, Egidijus Kazanavičius, Agnius Liutkevičius, Audronė Janavičiūtė

**Affiliations:** 1 Geriatric Department, Medical Academy, Lithuanian University of Health Sciences, 44307 Kaunas, Lithuania; gytedamu@gmail.com (G.D.); jurgaknasiene@gmail.com (J.K.); 2 Centre of Real Time Computer Systems, Kaunas University of Technology, 51424 Kaunas, Lithuania; egidijus.kazanavicius@ktu.lt (E.K.); agnius.liutkevicius@ktu.lt (A.L.); audrone.janaviciute@ktu.lt (A.J.)

**Keywords:** older adults, geriatric in-patients, technology, falls

## Abstract

*Background and objective*: The successful adoption of technology is becoming increasingly important to functional independence and successful ageing in place. A better understanding of technology usage amongst older people may help to direct future interventions aimed at improving their healthcare. We aimed to obtain the first data regarding technology use, including gerontechnologies, represented by fall detectors, from older adults in Lithuania. *Material and methods*: The research was carried out in the framework of the project Smart Gerontechnology for Healthy Ageing, which involved assessing the use of technologies and the readiness to use gerontechnologies, as represented by fall detectors. A total of 375 individuals that were more than 60 years of age were enrolled in the study. The self-reporting questionnaires were completed by geriatric in-patients, hospitalized in the geriatric department, and also by community-dwelling older adults. *Results*: Geriatric in-patients’ use of computers and the internet was associated with age (every year of age decreased the probability of computer and internet use by 0.9-times) and a positive attitude towards new technologies—this predictor increased the use of a computer by six-times in comparison with people who did not have such an attitude. Sex and education had no influence on computer use for geriatric in-patients. For community-dwelling older adults, the use of computers and internet was associated with age, education (a university education increased the use of computers and the internet by four times), and a positive attitude towards technologies. *Conclusions*: Lithuanian older women in the study used computers, the internet, and cell phones equally with men. Increasing age was a strong negative predictor of technology use. A positive attitude to new technologies was a strong positive predictor of technology use. Most geriatric patients and community-dwelling older adults were ready to use technologies that permit ageing in place.

## 1. Introduction

The lifespan of older people is continuously growing together with the proportion of older people. However, increased longevity is associated with the increased prevalence of diseases and injuries [1,2]. Instead of increasing the number of nursing institutions, it is important to develop services that can be applied in homes and within society. Ageing in place is a preference of many older adults. People want to spend their life in an environment that is familiar, comfortable, and manageable [3]. Such new type of services, which increase the independence of older people and permits them to age in place, are provided by gerontechnology [4,5]. The successful adoption of technology is becoming increasingly important to functional independence [6]. The hope is that, with the help of technologies, this can be achieved with high levels of efficiency, potentially reducing the individual and societal costs of caring for elderly people [7]. A better understanding of technology usage amongst older people may help to direct future interventions aimed at improving their healthcare. In various countries, numerous studies were performed that showed the increased use of technologies by this segment of the population [8,9]. However, the usage of technologies in Lithuania, including gerontechnologies, has not been investigated until now. Existing controversies in the country have given rise to the possibility that the data would differ from other European countries. On the one hand, pensions in Lithuania are amongst the lowest in the EU (mean pension is 287 Euro (2017) and the risk of pensioner poverty is 30.6 percent [10], thus, financially limiting access to technologies). On the other hand, for six years, Lithuania was a leader in Europe in the development of the fast fiber-optic internet (first position in Europe, eighth in the world, 2015) [11]. Our investigation was performed in the framework of the Smart Gerontechnology for Healthy Ageing project. The objective of the project is to create a new prototype of a health monitoring system for older people, which can be used in hospitals and at home. In the first stage of the project, it was important to assess the local situation of technology use in older adults, which has not been investigated in Lithuania until now. This can be very different from country to country depending on the information and communication technology infrastructure and economic wealth status of a region, as has been shown in the SHARE project investigating internet use in 17 European countries [12]. Moreover, it was important to obtain knowledge about which technologies are frequently used, and, therefore, have the possibility for use with incorporated software for health monitoring. 

Therefore, we aimed to obtain the first data regarding the use of technologies, including gerontechnologies, represented by fall detectors, by older adults in Lithuania, with the hypothesis based on the literature [13] that the likelihood to use technologies would be higher for those who were younger, male, more educated, and more interested in new technology. In addition, knowledge about the existence of technology that helps older people to stay longer at home, and the readiness to use it, was assessed.

## 2. Materials and Methods

The research was carried out as an exploratory research project, assessing the use of technologies and the readiness to use gerontechnologies, represented by fall detectors. A total of 375 individuals of more than 60 years of age were enrolled in the study. The study was approved by the regional bioethics committee at the Lithuanian University of Health Sciences (No. BE-2-26).

Self-reporting questionnaires were completed by geriatric in-patients, hospitalized in the geriatric department. Every third patient newly arrived in the geriatric department, 60 years of age and older, during the period from 1 April to 30 July 2017, was interviewed. Of the in-patients, 123 participated: 93 females, 30 males, with a mean age of 78.2 ± 9.3 years (140 patients were invited to participate, 17 met the exclusion criteria, and the participation rate was 88.9 percent). Exclusion criteria were a heavy state (inability to speak) and a heavy cognitive impairment (Mini Mental State Exam score <10) [14]. 

Self-reporting questionnaires were also completed also by community-dwelling persons (218 females and 34 males, aged 60 years and older, with a mean age 69.6 ± 5.6 years), who were attending Third Age University during the same period of time. None refused to complete the questionnaire, thus, the participation rate was 100 percent. 

The patient data was collected using questionnaires. Demographic variables collected during the interview included age, sex, and education level. Information on the use of technologies, knowledge, and the readiness to use technologies, including fall detectors, was collected. Information was collected on the use of older generation electronic devices (e.g., refrigerator, vacuum cleaner, washing machine) and newer digital technologies (computer, the internet, cell phones, fall detectors).

Study subjects were asked about their attitude toward new technologies (e.g., do you like to try new technologies?). They were also asked about their knowledge and readiness to use gerontechnologies:

1. Do you know about the technical means by which permit older people to feel safe and well in their own homes, preventing moving to homes for the elderly?
2. Do you know that there are technical means that can warn you before an imminent fall?
3. Do you know about fall detectors that can send an S.O.S signal about your fall to a family member or caregiver?
4. Would you like to try these means if you were asked?

Answers could be just “yes” or “no”.

Fall detectors are an example of gerontechnology that is easy to understand. In real life, none of the study subjects used the fall detectors. They were only asked about their readiness to use them as a sort of gerontechnology that permits them to age in place. 

The questionnaires were completed by geriatric in-patients (asking about the usage of technologies before hospitalization) and community-dwelling older adults (about the usage of technologies at home).

All participants were interviewed by trained research staff, and the interviews were standardized primarily with closed-ended questions. 

Statistical analysis was performed using the statistical software package SPSS 20.0 for Windows. Characteristics of the participants were compared using Pearson’s chi-square test, Fisher’s exact test, and the Student’s *t*-test. A two-tailed *P* value of <0.05 was considered statistically significant. Monte Carlo correction was applied with a variable dispersion over 25 percent. Multiple logistic regression analysis was performed by assessing the predictors of technology use. Predictors of technology use included in the regression analysis were age (years), sex (0- male, 1- female), education (1 – university, 0 – less than university), and positive attitude towards technologies (1).

## 3. Results

We compared the data of hospitalized geriatric patients and the data of the older population living in the community and attending Third Age University (Table 1), with the expectation that the data of the majority of older people would stay within these two extremes. Respondents living in the community used refrigerators, cell phones, computers, and the internet more often than geriatric in-patients. They also knew about the possibilities of gerontechnologies, such as fall detectors, and they were more inclined to use the new technologies, including fall detectors, than geriatric in-patients. Of the geriatric in-patients, 24.2 percent knew about technologies which would permit them to stay longer at home and not move to residential care, compared to 59.5 percent of community-dwelling older adults (*p* < 0.001). The willingness to try these technologies was expressed by 53.7 percent of geriatric in-patients and 84.5 percent of community-dwelling older adults (*p* < 0.001).

Preliminary data analysis showed that women tended to use technologies as actively as men. Cell phones were used by 93.7 percent of older women and 86.8 percent of older men (*p* = 0.089), computers were used by 65.5 percent of older women and 52.8 percent of older men (*p* = 0.087), whilst the internet was used by 64.3 percent of older women and 47.2 percent of older men (*p* = 0.021).

With regard to age groups, technologies were more often used by young-olds (i.e., 60–74 yrs) than by old-olds (75+ yrs). In the young-old group, 96.6 percent of seniors used cell phones and in the old-old group 81.4 percent used cellphones. Similarly, computer use was 73.6 percent and 36.0 percent, respectively, and internet use was 73.1 percent and 30.2 percent, respectively (*p* in all cases <0.001).

Computers at home were used by 20.3 percent of geriatric in-patients and 71.4 percent of community-dwelling older adults, *p* < 0.001. Multiple logistic regression analysis showed (Table 2) that the geriatric in-patients’ use of computers was associated with age (every year of age decreased the probability of computer use by 0.9-times) and a positive attitude towards new technologies—this predictor increased the use of computers by six-times in comparison to people who did not have such an attitude. Sex and education had no influence on computer use for geriatric in-patients. For community-dwelling older adults, the use of computers was associated with age, education (university education increased the use of computers by four-times), and a positive attitude towards technologies.

The internet was used by 16.3 percent of geriatric in-patients and 69.8 percent of community-dwelling older adults. Moreover, 13 persons (6.3 percent) of those who used computers, did not use the internet. For geriatric in-patients, the predictors of internet use were age (negative predictor—every year of age decreased the probability of computer use by 0.9-times) and a positive attitude towards new technologies (positive predictor, which increased internet usage by 10-times) (Table 3). For community-dwelling older adults, a negative predictor of internet use was age, and the positive predictors were university education and a positive attitude towards new technologies, increasing internet use by three-times.

Multiple logistic regression analysis of cell phone use showed that the same factors that influenced the use of computers and the internet did not predict cell phone use by community-dwelling older adults, that is, age, education, and a positive attitude towards new technologies were not associated with the use of cell phones (Table 4).

## 4. Discussion

Decline of physical and cognitive functions due to aging and sickness can be partially compensated using assistive and health technologies. Consequently, old people can stay for longer in their own homes [14]. The use of technology by older adults can not only help to satisfy their needs, but it also has the potential to give support to caregivers and to reduce health and social care costs [15]. The preference of older people is to age in place. Ageing-in-place, supported by the use of technologies, has a positive impact on the quality of life of older people, and it permits the usage of health and social care resources more effectively [16,17]. 

The aim of this study was to obtain data about technologies used by older people in Lithuania, and their ability to use modern IT technologies. Our study was not planned like an epidemiological study. Rather, it was an exploratory study, aiming to obtain the first data regarding technology use by seniors, as well as to consider the perspectives on gerontechnology in Lithuania. Obtaining this data is important for future planning in the creation of health monitoring systems that can be used at home and in the community, which may possibly be produced at a lower price than foreign analogues, and, therefore, will be more accessible to Lithuanian older people. The usage of technologies, including gerontechnologies, had not been investigated in Lithuania until now.

It was no surprise that people attending Third Age University more often knew about the existence of technologies permitting them to stay in their own home instead of moving to a nursing home, when compared to geriatric in-patients. On the other hand, most respondents agreed to use technologies if they needed them, and if these technologies would be accessible (i.e., not too expensive or available free of charge).

As an example of gerontechnology, fall detectors were used in our study. Older adults are the age group at the highest risk of falls and their subsequent complications. Consequences of a fall usually depend on the time spent on the floor or on the ground, that is, a prolonged stay increases the risk of complications [18,19]. Especially dangerous is a “long-lie” event when a person spends one hour or more on the floor as a result of a fall. A “long-lie” event is associated with serious injuries, higher mortality rates, and nursing home placement [20]. To prevent this and to ensure immediate assistance early fall detection is needed. This can be achieved through the use of fall detectors, which can be one of the means to permit seniors to age in place. Fall detectors can be separate devices or they can be incorporated into any health monitoring system.

According to the data of our study, most older adults (but not all of them) used common household equipment like refrigerators, vacuum cleaners, or washing machines. In community-dwelling older adults, 71.4 percent used computers and 69.8 percent used the internet. These numbers did not differ significantly from the prevalence of technology use by older adults in most developed countries [21]. On the other hand, only a minority of geriatric in-patients were using computers and the internet. Nevertheless, over 80 percent used cell phones. Technologies with software incorporated into cell phones are promising, because cell phones are widely used by geriatric in-patients and by older adults living in the community. The study subjects were interviewed about the use of cell phones with no distinction between simple mobiles and smart phones. The earliest generation of mobile phones can only make and receive calls. Nowadays, such simple cell phones have the possibility of internet use and a camera. The use of cell phones gives older adults and broad segments of the population better access to technology compared to previous technologies [22,23]. Older adults are more likely to own a cell phone than a desktop or laptop computer [9]. On the other hand, devices like cell phones are not stigmatizing to the individual using them—and that is important for users [24,25,26]. Individuals also want to keep control over devices to maintain their privacy and not to feel like they are constantly being monitored [24]. Noteworthy is that the stronger their health needs are, the less important are their privacy concerns when considering smart home technology [27,28]. 

Studies in other countries have shown that older individuals who use modern technologies are more likely to be younger, better educated, male, and more interested in new technology [9,29]. This was the reason to include these factors in the multiple logistic regression analysis.

According to our data, males did not use a computer, the internet, and cell phones more often than women. This was valid in both community-dwelling older adults and geriatric in-patients. This Lithuanian phenomenon can be explained by the use of IT technologies mostly for communication. Older women are communicating with their family members and friends, including those working abroad, no less actively than men. Usually women are the kin keepers of the family. According to official Lithuanian statistics, only 4.4 percent of older adults are buying in internet shops or ordering services using the internet [30].

Our data showed that age was a strong negative predictor of technology use. Every year of age decreased the probability of computer or internet use by 0.9-times. Thus, there is no surprise that there was a big gap in the usage of technologies between the two age groups of older adults (i.e., those aged 60–74 years, and those who were 75 and above). In this aspect, these two groups more closely resembled two different generations rather than close age groups. Actually, the older age group represented senior Lithuanians who grew up before the Second World War, and the representatives of people aged 60–74 years represented the generation born after the War, during Soviet occupation.

University education was a strong predictor of computer and internet use for community-dwelling older adults (*p* < 0.000), though this was not valid for geriatric in-patients. For geriatric in-patients, a strong positive predictor was a positive attitude towards new technologies. A positive attitude towards new technologies is important in computer and internet use, both by geriatric in-patients and by community-dwelling older adults.

Multiple logistic regression analysis of cell phones use showed that the same factors that influenced the use of computers and the internet were not valid for cell phone use by community-dwelling older adults, that is, age, education, and a positive attitude towards new technologies were not associated with the use of cell phones. We can only suggest that more important for cell phone use were other factors, like economic or health issues, which were not investigated in this study.

## 5. Conclusions

Lithuanian older women in the study used computers, the internet, and cell phones equally with men. Increasing age was a strong negative predictor of technology use. A positive attitude towards new technologies was a strong positive predictor of technology use. Most geriatric in-patients and community-dwelling older adults were ready to use technologies permitting them to age in place.

## 6. Limitations of the Study

The main limitation was that our study was not an epidemiological survey representing the entire older population of Lithuania, but rather it was an exploratory study aimed at gathering the first approximate data on the readiness of Lithuanian older adults to use technologies. Therefore, it restricts the generalizability of our findings.

## Figures and Tables

**Table 1:** Use of technologies by geriatric in-patients and older adults living in the community.

Variables	Geriatric in-Patients *n* = 123	Community-Dwelling Older Adults *n* = 252	*P*-Value
1. Use of technologies	*n* (%)	n (%)	
1.1. Refrigerator	119 (96.7)	251 (99.6)	0.042
1.2. Vacuum cleaner	103 (83.7)	227 (90.1)	0.076
1.3. Washing machine	105 (85.4)	231(91.7)	0.061
1.4. Cell phone	100 (81.3)	242 (96.4)	<0.001
1.5. Computer	25 (20.3)	180 (71.4)	<0.001
1.6. Internet	20 (16.3)	176 (69.8)	<0.001
2. Positive attitude towards new technologies	47 (38.2)	186 (73.8)	<0.001
3. Knows about technologies permitting them to stay longer in own home	30 (24.2)	150 (59.5)	<0.001
4. Knows about fall detectors	18 (14.6)	78 (31.0)	<0.001
5. Knows about the S.O.S signal	19 (15.6)	98 (38.9)	<0.001
6. Knows that they can be warned before a fall	20 (16.4)	80 (31.7)	0.002
7. Would use technologies if offered	66 (53.7)	213 (84.5)	<0.001

P: geriatric in-patients compared to community-dwelling older adults.

**Table 2:** Computer use by older adults: multiple logistic regression data.

Geriatric in-Patients *n* = 123				
	Odds Ratio	95.0 % CI		*P*-Value
		Lower	Upper	
Age	0.925	0.925	0.976	<0.005
Sex	1.125	0.335	3.775	0.850
Education	0.548	0.077	3.881	0.547
Positive attitude	6.357	2.174	18.590	<0.001
Constant	37.252			0.101
**Community-dwelling older adults *n* = 252**				
Age	0.889	0.836	0.945	<0.000
Sex	0.839	0.346	2.033	0.698
Education	4.106	2.121	7.948	<0.000
Positive attitude	3.412	1.769	6.582	<0.000
Constant	2749.000			<0.001

Variables: Age (years), sex (female-1), education (university-1), and positive attitude towards new technologies-1.

**Table 3:** Internet use by older adults: multiple logistic regression data.

Geriatric in-Patients *n* = 123				
	Odds Ratio	95.0 % CI		*P*-Value
		Lower	Upper	
Age	0.913	0.860	0.970	0.003
Sex	1.612	0.371	7.015	0.524
Education	0.704	0.088	5.623	0.740
Positive attitude	10.389	2.670	40.419	<0.001
Constant	33.341			0.156
**Community-dwelling older adults *n* = 252**				
Age	0.888	0.836	0.943	<0.000
Sex	0.908	0.385	2.141	0.825
Education	3.464	1.838	6.532	<0.000
Positive attitude	3.300	1.729	6.297	<0.000
Constant	2846.148			<0.000

Variables: Age (years), sex (female-1), education (university-1), and positive attitude towards new technologies-1.

**Table 4:** Cell phone use by older adults: multiple logistic regression data.

Geriatric in-Patients *n* = 123				
	Odds Ratio	95.0 % CI		*P*-Value
		Lower	Upper	
Age	0.925	0.857	1.000	0.049
Sex	1.459	0.498	4.271	0.491
Education	0.613	0.106	3.547	0.585
Positive attitude	4.533	1.215	16.906	0.024
Constant	1157.675			0.027
**Community-dwelling older adults *n* = 252**				
Age	0.934	0.825	1.057	0.277
Sex	1.347	0.249	7.298	0.730
Education	3.243	0.649	16.197	0.152
Positive attitude	0.670	0.128	3.499	0.635
Constant	2436.245			0.110

Variables: Age (years), sex (female-1), education (university-1), and positive attitude towards new technologies-1.
